# Identifying Frail-Related Biomarkers among Community-Dwelling Older Adults in Japan: A Research Example from the Japanese Gerontological Evaluation Study

**DOI:** 10.1155/2018/5362948

**Published:** 2018-01-22

**Authors:** N. Cable, A. Hiyoshi, N. Kondo, J. Aida, H. Sjöqvist, K. Kondo

**Affiliations:** ^1^Department of Epidemiology and Public Health, University College London, London, UK; ^2^Clinical Epidemiology and Biostatistics, School of Medical Sciences, Örebro University, Örebro, Sweden; ^3^Department of Health and Social Behavior, School of Public Health, University of Tokyo, Tokyo, Japan; ^4^Division of International and Community Oral Health, Graduate School of Dentistry, Tohoku University, Miyagi, Japan; ^5^Department of Statistics, Örebro University, Örebro, Sweden; ^6^Center for Preventive Medical Science, Chiba University, Chiba, Japan

## Abstract

We examined correlating clinical biomarkers for the physical aspect of frailty among community-dwelling older adults in Japan, using Japanese Gerontological Evaluation Study (JAGES). We used information from the JAGES participants (*N* = 3,128) who also participated in the community health screening in 2010. We grouped participants' response to the Study of Osteoporotic Fracture (SOF) Frailty Index into robust (=0), intermediate frail (=1), and frail (=2+) ones to indicate physical aspect of frailty. Independent of sex and age, results from multinomial logistic regression showed above normal albumin and below normal HDL and haemoglobin levels were positively associated with intermediate frail (RRR = 1.99, 95% CI = 1.22–3.23; RRR = 1.36, 95% CI = 1.33–1.39; RRR = 1.36, 95% CI = 1.23–1.51, resp.) and frail cases (RRR = 2.27, 95% CI = 1.91–2.70; RRR = 1.59, 95% CI = 1.51–1.68; RRR = 1.40, 95% CI = 1.28–1.52, resp.). Limited to women, above normal Hb1Ac level was similarly associated with intermediate frail and frail cases (RRR = 1.18, 95% CI = 1.02, 1.38; RRR = 2.56, 95% CI = 2.23–2.95, resp.). Use of relevant clinical biomarkers can help in assessment of older adults' physical aspect of frailty.

## 1. Introduction

Frailty has been regarded as manifesting multidimensional features of accelerated decline in cognitive and physical function and reducing reserve capacity in musculoskeletal, neurological, nutritional, and aerobic systems and resistance to stressors in the older population [1], which can lead older adults to disability [1] and mortality [2]. 

Of existing numerous frailty measures [3], many often rely on measuring physical function with some being less likely to be readily available in clinical settings and hence have limited utility [4]. Common approaches to capture the physical aspect of frailty such as accumulation of deficits suggested by Rockwood and a phenotype-based approach by Fried [5, 6] are not exceptional, requiring the use of special equipment or lengthy questions for the assessment [6].

On the other hand, the Study of Osteoporotic Fractures (SOF) Frailty Index which also takes the phenotype approach employs only three self-reported items [6] of muscle strengths, low energy, and unintentional weight loss. Frailty identified through this tool has been associated with falls, disability, and death [7, 8] or fracture [8] which has been regarded as a useful screening tool for the physical aspects of frailty at the population level [6]. 

Substantial protein intake among older adults appeared to be protective of physical functional loss that led to frailty among older adults [9, 10], despite limited research evidence to identify a definitive biomarker to capture this aspect of frailty [11]. Curcio and others [12] suggested impaired function in protein synthesis and regeneration in a human body could be indicated by the level of albumin, creatinine, uric acid, and haemoglobin. Additionally, sarcopenia that manifests loss of muscle mass and function has been found to be triggered by disturbance in protein synthesis [12] as well as impaired inflammatory function [13].

Frailty and sarcopenia are likely to be highly related [14] and we think irregularities in protein profile with frailty could be observed in biomarkers. Moreover, sarcopenia has been linked to metabolic and hormonal profiles [15]. Harada et al. [16] reported that study participants with sarcopenia had lower serum albumin level as well as BMI. Sarcopenia being portrayed as a profile progressing into frailty [15], loss of muscle mass, ectopic fat, low levels of oxygen consumption, and insulin resistance was thought to be highly related to the physical aspect of frailty. We think that higher Hb1Ac, LDL, and triglyceride could be reflective of the physical aspect of frailty indicated by the SOF Frailty Index, while lower albumin, haemoglobin, and HDL would be associated with the same aspect of frailty.

Japan regularly implements community-level health screening (=*“kenshin”* in Japanese) and collects clinical biomarkers such as protein, lipids, and blood glucose levels to assess individual health status [17]. Our aim is to identify correlating clinical biomarkers for the physical aspect of frailty that are regularly collected through community health screenings in Japan. This would be useful for identifying frailty (i.e., physical aspect) cases among community-dwelling older adults in a timely manner, as well as being an effective use of public resources.

## 2. Materials and Methods

### 2.1. Data

We used the Japanese Gerontological Evaluation Study (JAGES) and the health screening data, both collected during 2010 and 2011. JAGES is an ongoing ageing panel study, investigating factors associated with physical and psychological functional decline among older adults aged 65 and over living in Japan with no certified status of dependency (=being eligible for long-term care insurance benefits) [18–20]. Data were collected from over 110,000 participants across 31 municipalities; of these, health screening data were obtained from the participants from 6 municipalities. About half of the participants from these municipalities (*N* = 9,457) were linked with the health screening data, of which we used the data from 3,128 participants that had all relevant information.

The Ethics Committee on Research of Human Subjects at Nihon Fukushi University (number 10-05) approved the project, and the process of obtaining participants' consent for the use of their health screening data has been described elsewhere [19].

### 2.2. Measurements

We used albumin, creatinine, uric acid and haemoglobin, presence of protein in urine, high-density lipoprotein (HDL), low-density lipoprotein (LDL), triglyceride, and glycated haemoglobin (Hb1Ac) as explanatory variables. We also included the estimate of glomerular filtration rate (eGFR, derived through creatinine values) to elaborate findings on protein profiles in relation to the participants' kidney function.

Apart from the test results for proteinuria, all values were grouped into the following: below normal values, within the normal range, and above normal values based on the sex and, where possible, specific to age [21–23] (Supplementary Table 1). Presence of protein in urine was coded in a dichotomous manner, 1 being absence of protein (=normal) and 2 being trace or positive for protein. In addition, eGFR was coded into below normal and normal.

### 2.3. Outcome Measure

The SOF Frailty Index identifies the frail (i.e., physical aspect) elderly based on the assessment of three features: weight loss, inability to rise from a chair five times without using arms, and reduced energy level. Based on the definition we used the closest information that reflects these features. They were as follows: (1) having lost weight by 2-3 kg or more during the past six months; (2) being incapable of rising from chair without holding onto anything; and (3) not feeling full of energy. Summing up those dichotomous responses (0 = no, 1 = yes), the score was grouped into robust (=0), intermediate frail (=1), and frail (=2 and 3). As validation, we examined the association of this score on experience of falls among those who were in the baseline data (*N* = 66,609) but not from the six municipalities to minimise introducing a bias; we found strong and significant correlations between frailty and the frequencies of falls experienced in the previous year (Supplementary Table 2).

### 2.4. Statistical Analysis

Not meeting the proportional odds assumption, we applied multinomial logistic regression to test the associations between selected biomarkers and frailty, taking the robust group as the reference category. In our analyses, all estimates were adjusted for sex and age, while the cluster robust sandwich estimator was used to calculate standard errors to account for possible data clustering within municipalities. For Hb1Ac, sex-specific estimates for this value were provided as the association differed by sex.

## 3. Results

The mean age of the study participants was 70.6 years (SD = 5.3, range 65–97), while gender composition was similar (women = 52.3%). Of 3,128 participants, 41% were intermediate frail cases, while 13% were classified as frail (Table 1). Overall, more men were in the robust group, while more women were in the intermediate frail and frail groups, finding significant gender difference in distribution of frailty cases (*χ*^2^(2) = 27.36, *p* < 0.001).

Results from multinomial logistic regression provided partial support for the associations between clinical biomarkers and the physical aspect of frailty (Table 2). In the fully adjusted model (Model 2), above normal albumin and below normal haemoglobin and HDL levels were positively associated with intermediate frail cases (RRR = 1.99, 95% CI = 1.22–3.23; RRR = 1.36, 95% CI = 1.33–1.39; RRR = 1.36, 95% CI = 1.23–1.51, resp.). In a graded manner, these markers were also associated with frail cases (RRR = 2.27, 95% CI = 1.91–2.70; RRR = 1.59, 95% CI = 1.51–1.68; RRR = 1.40, 95% CI = 1.28–1.52, resp.).

In contrast to the positive association between above normal albumin level and the physical aspect of frailty, below normal albumin level was also positively associated with frail cases (RRR = 2.06, 95% CI = 1.48–2.87). Above normal HDL appeared to be protective of frail cases (RRR = 0.48, 95% CI = 0.26–0.89), with which below normal triglycerides had a similar association (RRR = 0.66, 95% CI = 0.47–0.93), but not with below normal LDL—that association was positive (RRR = 1.79, 95% CI = 1.21–2.64).

Despite the presence of positive associations between proteinuria and frail cases (RRR = 1.19, 95% CI = 1.05–1.36), this was not reflected on biomarkers related to kidney function, such as creatinine, uric acid, or eGFR. Above normal serum creatinine levels had a negative association with frail cases, while above normal uric acid or eGFR did not show significant associations with any statuses of the physical aspect of frailty.

Above normal Hb1Ac also showed similar association patterns with the physical aspect of two frailty statuses (RRR = 1.18, 95% CI = 1.02, 1.38; RRR = 2.56, 95% CI = 2.23–2.95, resp.), yet this was limited to females. Below normal Hb1Ac was also positively associated with intermediate frailty in women. Hb1Ac was not significantly associated with any of the physical aspects of frailty statuses among men.

## 4. Discussion

In our study, women were more likely to be physically frail than men. We found significant associations between above normal levels of albumin and below normal levels of haemoglobin and the physical aspect of frailty in a graded manner, suggesting that these markers could be candidate clinical biomarkers for monitoring the physical aspect of frailty in a community setting. Additionally, above normal Hb1Ac level was also positively associated with the physical aspect of frailty in women. At the same time, below normal albumin and LDL levels were positively associated with the physical aspect of frailty.

### 4.1. Comparison with Other Studies

Low serum albumin level is likely to be indicative of cognitive frailty in the elderly [24], which led to our hypothesis supported by finding a similar association between normal albumin level and the physical aspect of frailty. Unexpectedly we also found that high serum albumin levels were positively associated with the physical aspect of frailty in a graded manner. However, albuminuria was found to be positively associated with frailty [25] which could offer an explanation for this association.

Despite the lack of consensus in associations between biomarkers and frailty in previous studies [11], a moderate link between haemoglobin and frailty [26] was reported, while there was a reported link between low HDL level and ADL-related disability [27]. Supporting our hypothesis, the associations between these markers and the physical aspect of frailty extend additional support to these existing findings.

In our study, below normal HDL and triglyceride levels were associated with the physical aspect of frailty in a complementary manner, which supported our hypothesis; however, we unexpectedly found that below normal levels of LDL appeared to be a risk for the physical aspect of frailty. Strong research evidence to link low LDL and mortality among elderly adults [28] could explain this association as a possible ageing process. Similarly, the negative association between above normal creatinine and the physical aspect of frailty could be due to the ageing process, as the value closely relates to muscle mass [29].

We expected to find a positive association between proteinuria and the physical aspect of frailty, yet the support did not extend to another kidney function indicator, eGFR that was not consistent with the finding reported by Ballew et al. [25]. However, it was reported that the association between creatinine-based eGFR and frailty was moderate, compared to the association between cystatin C-based eGFR and frailty, which was found to be more linearly associated with physical function among older adults compared to creatinine-based eGFR [30]. We used serum creatinine to derive participants' eGFR. Associations between serum creatinine and uric acid and the physical aspect of frailty were inconclusive; however, creatinine-based eGFR may not be accurately capturing study participants' kidney function. On the other hand, a positive association between albuminuria and frailty reported by Ballew et al. [25] can be an alternative explanation for finding the association between proteinuria and frail cases and between above normal serum albumin level and the physical aspect of frailty. Given that our study is cross-sectional, participants' protein intake can be related and further research is needed to determine whether the association of the high circulation of protein in the body of frail adults is due to poor kidney function or a feature of protein synthesis and dysregulation accompanied with frailty.

Moreover, diabetes was found to be related to frailty [31], which could not support our hypothesis in men. We are unable to offer a possible explanation for this gender-specific association between Hb1Ac and the physical aspect of frailty among women, and this warrants further investigation.

### 4.2. Strengths and Weaknesses of the Study

In our study, we derived a frailty score using questions similar to those used for the SOF Frailty Index. Our frailty score was highly correlated with a fall during the last 12 months among JAGES participants who were not from the municipalities where the health screening data was provided (Supplemental Table 2) which offers validation to our frailty score.

JAGES participants are limited to those who are not certified with long-term nursing care insurance (=“yokaigo” in Japanese) and therefore they are likely to be healthy, while participating in health check-ups for older adults is voluntary. It is possible that our findings are limited to those who are relatively healthy and interested in health. Despite the limitation in our research setting, we found reasonable numbers of intermediate and frail cases and significant associations between some biomarkers and the physical aspect of frailty.

Japanese health check-ups do not collect conventional inflammatory markers such as C-reactive protein, interleukin-6, or hair cortisol that were found to be strongly associated with frailty [13]. We cannot and do not claim causality between the suggested links since the associations were sought in a cross-sectional manner. Nevertheless, graded associations between the three clinical biomarkers that are albumin, haemoglobin, and HDL and the physical aspect of frailty suggest that these clinical biomarkers can be useful for monitoring the changes in the physical aspect of frailty. Additionally, observing the level of Hb1Ac could be useful for monitoring the physical aspect of frailty among women.

### 4.3. Clinical Implications and Future Research

Unlike other frailty measures, application of the SOF Frailty Index does not require special equipment [6] and uses only three items which are fewer than Fried et al.'s [32]. Using the SOF Frailty Index potentially offers benefits for screening the physical aspect of frailty among older adults in community settings. The clinical biomarkers for this study were obtained from existing health screening data. We suggest that subjective assessment of physical frailty through the SOF Frailty Index could be complemented by incorporating existing information on albumin, haemoglobin, and HDL that are likely to be collected through health screening. 

In our study, we grouped values of biomarkers into three groups (=below normal, normal, and above normal) where possible, to be relevant and meaningful to clinical practices. Treating clinical values in this way, we were able to show how below normal (=lack of production) albumin, haemoglobin, and HDL and above normal (=possible accumulation of the metabolic wastes) albumin were uniquely associated with the physical aspect of frailty.

Maintaining reasonable musculoskeletal health is one of the preventive strategies suggested for healthy ageing by WHO [33]. Future studies that aim to investigate the progression between relevant biomarkers and the physical aspect of frailty would contribute to the existing knowledge by shedding light on the biological mechanism of the physical aspect of frailty among older adults. Preventive interventions for the physical aspect of frailty through promoting musculoskeletal health could be objectively assessed using protein- and lipid-related biomarkers, such as albumin, haemoglobin, and HDL.

Suggesting these clinical biomarkers as candidate markers for the physical aspect of frailty warrants specificity and sensitivity of these markers. Frailty is a syndrome, manifesting multiple features [1] and the cause of frailty is difficult to identify [34], suggesting that we need to take a comprehensive approach to understanding the biological process of frailty in general. Our findings are relatively new to the area and further studies are required to identify possible profiles of frailty in the physical aspect guided by relevant biomarkers.

## 5. Conclusion

Using albumin, haemoglobin, and HDL could effectively validate the physical aspect of frailty cases identified by the SOF Frailty Index in community-dwelling older adults. Longitudinal observation to monitor the physical aspect of frailty among this population can be objectively assessed through these clinical biomarkers along with self-report on that index.

## Figures and Tables

**Table 1:** Sample characteristics, numbers of participants, and proportions (%) by levels (below normal, normal, and above normal) of study biomarkers according to the SOF Frailty category (*N* = 3,128).

	SOF Frailty Index category		
	Robust	Intermediate	Frail
	(*n* = 1,434)	(*n* = 1,280)	(*n* = 414)
	*Frequency (row%)*		
*Demographic*			
Age in year, mean (SD)	70.9 (4.8)	71.7 (5.3)	73.6 (6.4)
Gender			
Men	732 (49.1)	609 (40.8)	151 (10.1)
Women	702 (42.9)	671 (41.0)	263 (16.1)
*Biomarkers (units)*			
HbA1c (%): men			
Below normal	81 (53.6)	53 (35.1)	17 (11.3)
Normal	527 (48.3)	454 (41.7)	109 (10.0)
Above normal	121 (48.8)	101 (40.7)	26 (10.5)
HbA1c (%): women			
Below normal	120 (40.4)	131 (44.1)	46 (15.5)
Normal	532 (44.8)	482 (40.6)	174 (14.6)
Above normal	53 (34.4)	59 (38.3)	42 (27.3)
HDL (mg/dL)			
Below normal	147 (38.3)	174 (45.3)	63 (16.4)
Normal	1255 (46.7)	1087 (40.4)	347 (12.9)
Above normal	32 (58.2)	19 (34.5)	4 (7.3)
LDL (mg/dL)			
Below normal	25 (45.5)	16 (29.1)	14 (25.5)
Normal	1306 (45.7)	1172 (41.1)	377 (13.2)
Above normal	103 (47.2)	92 (42.2)	23 (10.6)
Triglyceride (mg/dL)			
Below normal	5 (35.7)	8 (57.1)	1 (7.1)
Normal	1023 (46.4)	883 (40.1)	297 (13.5)
Above normal	406 (44.6)	389 (42.7)	116 (12.7)
Haemoglobin (g/dL)			
Below normal	259 (38.3)	297 (43.9)	120 (17.8)
Normal	1118 (48.0)	935 (40.1)	277 (11.9)
Above normal	57 (46.7)	48 (39.3)	17 (13.9)
Serum albumin (g/dL)			
Below normal	89 (36.0)	89 (36.0)	69 (27.9)
Normal	1335 (46.8)	1176 (41.2)	341 (12.0)
Above normal	10 (34.5)	15 (51.7)	4 (13.8)
Creatinine (mg/dL)			
Below normal	34 (43.6)	34 (43.6)	10 (12.8)
Normal	1273 (46.3)	1117 (40.6)	362 (13.2)
Above normal	127 (42.6)	129 (43.3)	42 (14.1)
Uric acid (mg/dL)			
Below normal	27 (45.0)	25 (41.7)	8 (13.3)
Normal	1252 (46.0)	1120 (41.1)	352 (12.9)
Above normal	155 (45.1)	135 (39.2)	54 (15.7)
Urine			
Protein absence	1213 (45.9)	1083 (41.0)	344 (13.0)
Protein: trace or positive	221 (45.3)	197 (40.4)	70 (14.3)
eGFR (mL/min/1.73 m^2^)			
Normal	1120 (47.0)	963 (40.4)	300 (12.6)
Above normal	314 (42.1)	317 (42.6)	114 (15.3)

**Table 2:** Relative risk ratio (RRR) with 95% CI in parentheses for intermediate frail and frail by biomarker levels among the JAGES study participants (*N* = 3,128).

Biomarkers	Robust	Intermediate frail		Frail	
		Model 1	Model 2^b^	Model 1	Model 2^b^
HbA1c (male)					
Below normal	Reference	0.85 (0.76, 0.96)	0.86 (0.73, 1.02)	1.28 (0.50, 3.30)	1.21 (0.46, 3.18)
Normal	Reference	1.00	1.00	1.00	1.00
Above normal	Reference	0.99 (0.72, 1.36)	0.99 (0.70, 1.39)	1.04 (0.75, 1.43)	0.99 (0.76, 1.29)
HbA1c (female)					
Below normal	Reference	1.19 (1.12, 1.26)	1.22 (1.13, 1.31)	1.21 (0.86, 1.72)	1.20 (0.81, 1.78)
Normal	Reference	1.00	1.00	1.00	1.00
Above normal	Reference	1.21 (1.07, 1.36)	1.18 (1.02, 1.38)	2.63 (2.22, 3.11)	2.56 (2.23, 2.95)
HDL					
Below normal	Reference	1.34 (1.20, 1.48)	1.36 (1.23, 1.51)	1.44 (1.29, 1.62)	1.40 (1.28, 1.52)
Normal	Reference	1.00	1.00	1.00	1.00
Above normal	Reference	0.70 (0.51, 0.96)	0.69 (0.46, 1.04)	0.50 (0.26, 0.97)	0.48 (0.26, 0.89)
LDL					
Below normal	Reference	0.74 (0.51, 1.09)	0.66 (0.41, 1.08)	2.34 (1.56, 3.50)	1.79 (1.21, 2.64)
Normal	Reference	1.00	1.00	1.00	1.00
Above normal	Reference	1.02 (0.68, 1.53)	1.03 (0.66, 1.60)	0.82 (0.63, 1.06)	0.94 (0.76,1.15)
Triglyceride					
Below normal	Reference	1.82 (0.69, 4.82)	2.09 (0.70, 6.26)	0.73 (0.51, 1.04)	0.66 (0.47,0.93)
Normal	Reference	1.00	1.00	1.00	1.00
Above normal	Reference	1.10 (0.99, 1.22)	1.05 (0.97, 1.14)	0.92 (0.74, 1.14)	0.88 (0.71,1.08)
Haemoglobin					
Below normal	Reference	1.34 (1.29, 1.39)	1.36 (1.33, 1.39)	1.75 (1.57, 1.96)	1.59 (1.51,1.68)
Normal	Reference	1.00	1.00	1.00	1.00
Above normal	Reference	1.02 (0.96, 1.09)	1.01 (0.96, 1.07)	1.27 (0.69, 2.34)	1.28 (0.81,2.02)
Serum albumin					
Below normal	Reference	1.06 (0.75, 1.48)	0.96 (0.68, 1.36)	2.49 (1.76, 3.52)	2.06 (1.48,2.87)
Normal	Reference	1.00	1.00	1.00	1.00
Above normal	Reference	1.85 (1.18, 2.88)	1.99 (1.22, 3.23)	2.07 (1.50, 2.85)	2.27 (1.91,2.70)
Creatinine					
Below normal	Reference	1.21 (0.61, 2.42)	1.27 (0.60, 2.66)	1.35 (0.66, 2.74)	1.32 (0.63,2.80)
Normal	Reference	1.00	1.00	1.00	1.00
Above normal	Reference	1.08 (1.02, 1.15)	0.96 (0.82, 1.12)	0.94 (0.79, 1.14)	0.71 (0.63,0.80)
Uric acid					
Below normal	Reference	1.09 (0.78, 1.51)	1.08 (0.86, 1.36)	1.30 (0.93, 1.80)	1.10 (0.75,1.59)
Normal	Reference	1.00	1.00	1.00	1.00
Above normal	Reference	0.90 (0.70, 1.15)	0.85 (0.65, 1.10)	0.92 (0.88, 0.96)	0.88 (0.77,1.02)
Urine					
Protein: absent	Reference	1.00	1.00	1.00	1.00
Protein: trace or positive	Reference	1.02 (1.00, 1.05)	1.02 (0.99, 1.04)	1.24 (1.08, 1.42)	1.19 (1.05,1.36)
eGFR					
Normal	Reference	1.00	1.00	1.00	1.00
Above normal	Reference	1.11 (1.04, 1.18)	1.12 (0.99, 1.26)	1.11 (0.98, 1.25)	1.17 (0.95,1.44)

Model 1: adjusted for age and sex for biomarkers other than HbA1c; adjusted for age for HbA1c. Model 2: Model 1 + all biomarkers. ^b^For Hb1Ac, sex interaction is included and estimates are provided by each gender. *Note*. A robust sandwich estimator for standard errors was used to adjust data clustering within municipalities.

## Supplementary Materials

Supplementary Materials Supplementary Table  1: clinical normal range utilised to group clinical biomarkers obtained from the Japanese study participants. Supplementary Table  2: validation of the score obtained from the Study of Osteoporotic Fractures (SOF) Frailty Index by testing an association with the previous experiences of fall among the study participants of JAGES 2010 who were not living in the municipalities with the health screening data (*N* = 66,609).
