# Supplementary material for: Identifying Frail-Related Biomarkers among Community-Dwelling Older Adults in Japan: A Research Example from the Japanese Gerontological Evaluation Study
Source: Biomed Res Int. 2018 Jan 22;2018:5362948. doi: 10.1155/2018/5362948 (PMC5828560; doi:10.1155/2018/5362948)
Supplement: Supplementary Materials — Supplementary Table 1: clinical normal range utilised to group clinical biomarkers obtained from the Japanese study participants. Supplementary Table 2: validation of the score obtained from the Study of Osteoporotic Fractures (SOF) Frailty Index by testing an association with the previous experiences of fall among the study participants of JAGES 2010 who were not living in the municipalities with the health screening data (N = 66,609). [file 5362948.f1.pdf]

Supplementary Table 1. Clinical normal range utilised to group clinical biomarkers obtained from the Japanese study participants.

Biomarkers (Unit)		
HbA1c (%)	Below normal	Male: <4.97; Female: <5.11
	Normal	Male: 4.97-6.03; Female: 5.11-6.20
	Above normal	Male: >6.03; Female: >6.20
HDL (mg/dL)	Below normal	Male: <38; Female: < 48
	Normal	Male: 38-90; Female: 48-103
	Above normal	Male: >90; Female: >103
LDL (mg/dL) [a]	Below normal	<65
	Normal	65-163
	Above normal	>163
Triglyceride (mg/dL)	Below normal	Male: < 39; Female: <32
	Normal	Male: 39-198; Female: 32-134
	Above normal	Male: >198; Female: >134
Haemoglobin (g/dL)	Below normal	Male: < 13.7; Female: <11.9
	Normal	Male: 13.7-16.4; Female: 11.9-14.6
	Above normal	Male: >16.4; Female: >14.6
Serum albumin (g/dL)	Lower abnormal	Male: <3.9; Female: <4.0
	Normal	Male: 3.9-4.7; Female:4.0-4.8
	Above normal	Male: >4.7; Female: >4.8
Creatinine (mg/dL)	Below normal	Male: <0.66; Female: <0.47
	Normal	Male: 0.66-1.08; Female: 0.47-0.82
	Above normal	Male: >1.08; Female: >0.82
Uric acid (mg/dL)	Below normal	Male: <3.6; Female: <2.6
	Normal	Male: 3.6-7.9; Female: 2.6-5.9
	Above normal	Male: >7.9; Female: >5.9
Urine	Protein: Negative	(-)
	Protein: Trace or Positive	(±,+, 2+, 3+, 4+)
eGFR ($ml/min/1.73m^2$)[b]	Below normal	<60
	Normal	≥60

[a] No sex-specific clinical normal range.

[b] Derived using the sex-specific formula: Male = 194 x $Creatinine^{-1.09}$ x $Age^{-0.28}$; Female = 194 x $Creatinine^{-1.09}$x $Age^{-0.28}$ -0.739

Supplement Table 2: Validation of the score obtained from the Study of Osteoporotic Fractures (SOF) Frailty Index by testing an association with the previous experiences of fall among the study participants of JAGES 2010 who were not living in the municipalities with the health screening data (N= 66,609).

	**Robust** (n=28,198)		**Intermediate** (n=27,380)		**Frail** (n=11,031)	
	**%**	**RRR (95%CI)**	**%**	**RRR (95%CI)**	**%**	**RRR (95%CI)**
**Fall**						
None	77.1	Reference	69.2	Reference	54.3	Reference
Once	19.1	Reference	24.0	1.38 (1.32- 1.42) ***	30.0	2.05 (1.96- 2.14)***
More than once	3.9	Reference	6.9	1.93 (1.78- 2.10) ***	15.7	5.19 (4.65- 5.79) ***

Notes: Estimates are adjusted for age, sex and residential clustering.
RRR: relative risk ratio
*p-value <0.05; **p-value < 0.01; ***p-value <0.001
